# Association of Early Antibiotic Exposure With Childhood Body Mass Index Trajectory Milestones

**DOI:** 10.1001/jamanetworkopen.2021.16581

**Published:** 2021-07-12

**Authors:** Izzuddin M. Aris, Pi-I D. Lin, Sheryl L. Rifas-Shiman, L. Charles Bailey, Janne Boone-Heinonen, Ihuoma U. Eneli, Anthony E. Solomonides, David M. Janicke, Sengwee Toh, Christopher B. Forrest, Jason P. Block

**Affiliations:** 1 Division of Chronic Disease Research Across the Lifecourse, Department of Population Medicine, Harvard Medical School and Harvard Pilgrim Health Care Institute, Boston, Massachusetts; 2 Applied Clinical Research Center, Department of Pediatrics, Children’s Hospital of Philadelphia, Philadelphia, Pennsylvania; 3 Department of Pediatrics, Perelman School of Medicine, University of Pennsylvania, Philadelphia; 4 School of Public Health, Oregon Health & Science University, Portland; 5 Center for Healthy Weight and Nutrition, Nationwide Children’s Hospital, Columbus, Ohio; 6 Center for Biomedical Research Informatics, NorthShore University Health System, Evanston, Illinois; 7 Department of Clinical and Health Psychology, College of Public Health and Health Professions, University of Florida, Gainesville; 8 Division of Therapeutics Research and Infectious Disease Epidemiology, Department of Population Medicine, Harvard Pilgrim Health Care Institute, Harvard Medical School, Boston, Massachusetts

## Abstract

**Importance:**

Past studies have showed associations between antibiotic exposure and child weight outcomes. Few, however, have documented alterations to body mass index (BMI) (calculated as weight in kilograms divided by height in meters squared) trajectory milestone patterns during childhood after early-life antibiotic exposure.

**Objective:**

To examine the association of antibiotic use during the first 48 months of life with BMI trajectory milestones during childhood in a large cohort of children.

**Design, Setting, and Participants:**

This retrospective cohort study used electronic health record data from 26 institutions participating in the National Patient-Centered Clinical Research Network from January 1, 2009, to December 31, 2016. Participant inclusion required at least 1 valid set of same-day height and weight measurements at each of the following age periods: 0 to 5, 6 to 11, 12 to 23, 24 to 59, and 60 to 131 months (183 444 children). Data were analyzed from June 1, 2019, to June 30, 2020.

**Exposures:**

Antibiotic use at 0 to 5, 6 to 11, 12 to 23, 24 to 35, and 36 to 47 months of age.

**Main Outcomes and Measures:**

Age and magnitude of BMI peak and BMI rebound.

**Results:**

Of 183 444 children in the study (mean age, 3.3 years [range, 0-10.9 years]; 95 228 [51.9%] were boys; 80 043 [43.6%] were White individuals), 78.1% received any antibiotic, 51.0% had at least 1 episode of broad-spectrum antibiotic exposure, and 65.0% had at least 1 episode of narrow-spectrum antibiotic exposure at any time before 48 months of age. Exposure to any antibiotics at 0 to 5 months of age (vs no exposure) was associated with later age (β coefficient, 0.05 months [95% CI, 0.02-0.08 months]) and higher BMI (β coefficient, 0.09 [95% CI, 0.07-0.11]) at peak. Exposure to any antibiotics at 0 to 47 months of age (vs no exposure) was associated with an earlier age (−0.60 months [95% CI, −0.81 to −0.39 months]) and higher BMI at rebound (β coefficient, 0.02 [95% CI, 0.01-0.03]). These associations were strongest for children with at least 4 episodes of antibiotic exposure. Effect estimates for associations with age at BMI rebound were larger for those exposed to antibiotics at 24 to 35 months of age (β coefficient, −0.63 [95% CI, −0.83 to −0.43] months) or 36 to 47 (β coefficient, −0.52 [95% CI, −0.72 to −0.31] months) than for those exposed at 0 to 5 months of age (β coefficient, 0.26 [95% CI, 0.01-0.51] months) or 6 to 11 (β coefficient, 0.00 [95% CI, −0.20 to 0.20] months).

**Conclusions and Relevance:**

In this cohort study, antibiotic exposure was associated with statistically significant, but small, differences in BMI trajectory milestones in infancy and early childhood. The small risk of an altered BMI trajectory milestone pattern associated with early-life antibiotic exposure is unlikely to be a key factor during prescription decisions for children.

## Key Points

**Question:**

Is early antibiotic exposure associated with altered body mass index (BMI) trajectory milestone patterns in childhood?

**Findings:**

This cohort study of 183 444 children identified small associations with age and magnitude of BMI at peak in infancy and rebound in early childhood after antibiotic exposure before 48 months of age.

**Meaning:**

The findings of this study suggest these small associations with BMI trajectory milestones after early-life antibiotic exposure should not affect the individual decision to prescribe antibiotics.

## Teaser

This cohort study uses data from the National Patient-Centered Clinical Research Network (PCORnet) Antibiotics Childhood Growth Study to examine the association of antibiotic use during the first 4 years of life and body mass index trajectory milestones during childhood in a large cohort of children.

## Introduction

Antibiotics are the most commonly prescribed medications in early childhood.^1^ Half of these prescriptions may be inappropriate,^2^ and antibiotic use has been linked with excess weight gain and risk of later childhood obesity.^3,4^ Most studies have found a small dose-response association with higher body mass index (BMI) *z* scores and odds of overweight or obesity in midchildhood with antibiotic exposure in early life (vs no exposure).^5,6,7,8,9^ It is hypothesized that antibiotics might affect energy metabolism and homeostasis through alterations to the gut microflora.^10,11^ Antibiotic use, therefore, may be a modifiable risk factor for later childhood obesity.

Prior studies that examined these associations^3,5,12,13,14,15^ have primarily focused on antibiotic exposures before 24 months of age and weight outcomes measured only on a single occasion. Few studies^16^ have investigated whether antibiotic exposure after 24 months of age is associated with greater weight or whether early antibiotic exposure alters longitudinal BMI trajectory patterns during childhood. An altered timing of child growth milestones (eg, later age at BMI peak in infancy and earlier age at BMI rebound in early childhood^17^) has been associated with excessive weight gain and cardiometabolic risk in later childhood.^18,19^ Examining these associations is important to assess whether antibiotic exposure across childhood could alter future weight and whether antibiotic exposure changes child growth milestones before reaching an end point. Coupled with prior studies on weight outcomes at fixed points in midchildhood, examining these intermediate milestones could provide information about whether antibiotic exposure might alter weight in the short and long term.

To address these gaps, we used data from the National Patient-Centered Clinical Research Network (PCORnet) Antibiotics Childhood Growth Study, a large, diverse multi-institutional study from a national research network that captured antibiotic prescribing data and anthropometrics obtained from clinical encounters.^20^ We examined dose-response associations of early antibiotic use before 48 months of age with longitudinal BMI patterns throughout childhood. We hypothesized that exposure to a greater number of episodes of antibiotic use in early life would be associated with altered BMI trajectory milestone patterns (ie, later age at BMI peak and earlier age at BMI rebound).

## Methods

### Cohort Formation

PCORnet is a collaborative network that includes data on approximately 75 large health systems in the US. Its purpose is to accelerate patient-centered outcomes research that uses both observational and experimental designs.^20^ Data from electronic health records at member health systems were extracted and transformed to a common data model to support data interoperability for research. Thirty-six participating health systems contributed data for the study of childhood antibiotic exposure and growth (683 485 children); these are grouped into 28 network partners because some institutions combined data into a centralized data warehouse. The institutional review boards of each institution approved the study, allowing for the transfer of deidentified patient-level data to Harvard Pilgrim Health Care Institute, where all statistical analyses were conducted. The institutional review board of the lead site (Harvard Pilgrim Health Care Institute) also approved the study as human subjects research with minimal risk and granted a waiver of informed consent. This study followed the Strengthening the Reporting of Observational Studies in Epidemiology (STROBE) reporting guideline.

**Figure 1.**
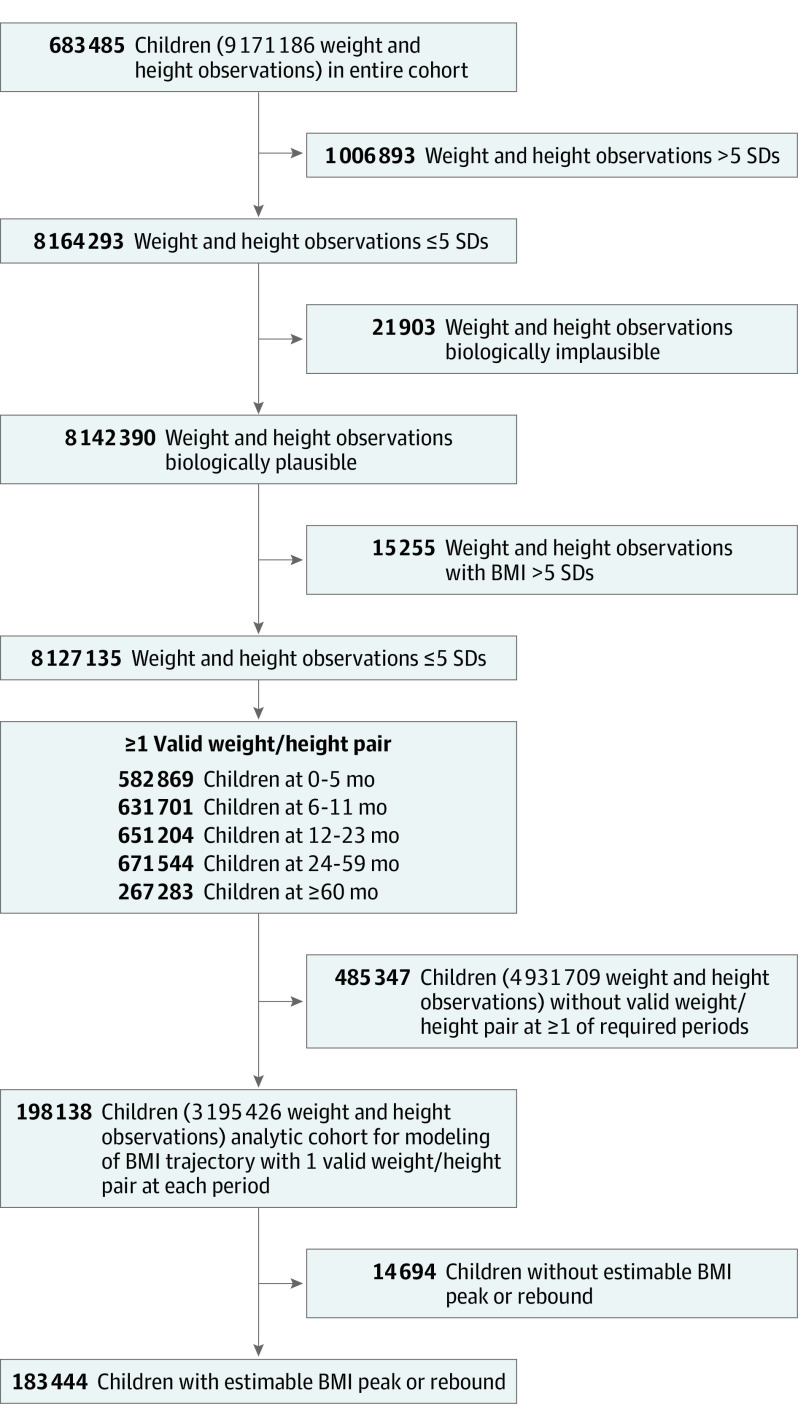
Flowchart for Analytic Cohort Formation BMI indicates body mass index.

We required children to have a valid record of their birth date and at least 1 pair of valid same-day height and weight measurements at each of the following age periods: 0 to 5, 6 to 11, 12 to 23, 24 to 59, and 60 to 131 months. We removed anthropometric measurements that were biologically implausible (ie, >5 SDs) using age- and sex-specific height and weight norms according to the Centers for Disease Control and Prevention reference growth charts.^21^ We additionally removed implausible measurements in height and weight trajectory data for each child using a conditional growth percentile approach, as detailed by Yang and Hutcheon.^22^ After removing implausible anthropometric measurements, our analytic cohort for modeling of BMI trajectories (Figure 1) included 198 138 children from 26 network partners with 3 195 426 height and weight observations (mean, 16 [range, 5-116] observations per child). Our final analytic sample included 183 444 children (92.6%) with estimable BMI peak and rebound and excluded 14 694 children (7.4%) with either no BMI peak (ie, showed no decline in BMI after the rise in infancy) or no BMI rebound (ie, showed no rise in BMI after the decline in early childhood). Data were collected from January 1, 2009, to December 31, 2016.

### Exposure

The PCORnet Antibiotics Childhood Growth Study group^5,20^ described the classification of antibiotic exposure for PCORnet in detail elsewhere. In brief, we constructed a set of terms for systemic antibiotics using the National Library of Medicine’s RxNorm terminology.^23^ During a manual review of the list of antibiotics, we excluded antiprotozoal medications, antibiotics not available in the US, veterinary medicines, and most intravenous medications. This restriction ensured consistency across the network—several did not have inpatient medications—and allowed this study to focus on antibiotic decision-making for mild to moderate outpatient infections and antibiotics with uses that are more modifiable than those of intravenous medications. The final antibiotic list included only oral and common intramuscular formulations (eg, ceftriaxone) likely to be prescribed in the outpatient or emergency department setting. We accounted for duplicated same-day prescriptions and created antibiotic treatment episodes by joining antibiotic prescriptions within 10 days, giving priority to the broadest-spectrum antibiotic prescribed. The PCORnet study group^24^ previously compared antibiotic prescription data from electronic health records with pharmacy dispensing records for antibiotics, which showed that misclassification of antibiotic exposure was low. We defined narrow-spectrum antibiotics as penicillin, amoxicillin, and dicloxacillin sodium and broad-spectrum antibiotics as all other antibacterials. We also created age-specific periods (0-5, 6-11, 12-23, 24-35, and 36-47 months) to examine potentially sensitive periods of antibiotic exposure.

### Age and Magnitude of BMI at Peak and Rebound

We used same-day height and weight measurements obtained at ages 0 to 5, 6 to 11, 12 to 23, 24 to 59, and 60 to 131 months to calculate BMI (as weight in kilograms divided by height in meters squared). To estimate BMI peak and rebound, we used statistical models rather than direct observations and visual inspection of individual BMI-for-age curves, which are often prone to large interobserver variation.^25^ We fitted child-specific BMI curves using mixed-effects models with natural cubic spline functions for age to capture the nonlinear trend in BMI and included random effects for each spline function to account for repeated measures in the same child. We applied constraints to increase stability of the curve by fixing the spline to be linear before 1 month and after 101 months. The fixed-effects component of the model was as follows:
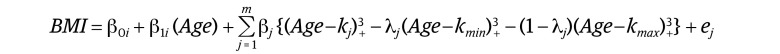
where for child *i*, *k_min_* and *k_max_* are boundary knots; *k_j_*, interior knot point *j* between boundary knots; *λ_j_*, the distance between knot points *k_m_* and *k_j_*; *m*, the number of interior knots between boundary knots; *j*, knot point 1, 2, …, *m*; *e*, error; and (*age* − *k_j_)*^3^_+_, age − *k* if age is greater than or equal to *k_j_*. Given the known differences in BMI trajectory patterns between boys and girls,^26^ we modeled sex-specific trajectories by including interactions of child sex with spline terms as fixed parameters in the model. The random-effects component of the model contained random effects for the intercept, linear age slopes, and spline functions.

We considered 2 approaches to select knot locations: at equally spaced percentiles from birth to 131 months of age and using knowledge of underlying biology of BMI growth patterns.^27,28^ We determined the optimal number (6 knots) and location (1, 6, 12, 24, 60, and 101 months) of knots using the bayesian information criterion. We assessed model fit using a residual plot of observed and predicted BMI (eFigure 1 in the Supplement). We estimated the age (in months) at peak and rebound by differentiation of child-specific BMI curves; the peak and rebound are located at ages where the derivative of the curve equals zero. We estimated the magnitude of BMI at peak and rebound as the highest and lowest points, respectively, of the child-specific BMI curve.

### Covariates

We selected covariates that were available in electronic health records and for which previous studies showed associations between antibiotic use and childhood growth.^3,4,5,8,12,13,14,16^ We categorized race as White, Black, Asian, other (Pacific Islander, American Indian, or Alaska Native), or unknown and Hispanic ethnicity using yes or no. We defined asthma as at least 2 asthma diagnosis codes before 72 months of age and preterm birth as any preterm diagnosis code before 24 months of age. We defined exposure to oral corticosteroids before 48 months of age, which is known to promote weight gain, as a categorical count of 0, 1, 2, 3, or 4 or more episodes. We defined the use of health care services (which could be associated with antibiotic prescriptions and child weight) as a categorical count (0, 1, 2, 3, or ≥4) of all clinical encounters before 48 months of age, including inpatient, emergency, or ambulatory visits. We determined the presence of complex chronic conditions (which could be associated with different growth patterns) using a list of conditions and corresponding codes as reported by Feudtner et al.^29^ We defined infection diagnoses using the approach of Fleming-Dutra et al,^30^ yielding categorical episodes (0, 1, 2, 3, or ≥4) classified as tier 1 (strong indication for antibiotics), tier 2 (possible improvement with antibiotics), and tier 3 (unlikely to benefit from antibiotics).

### Statistical Analysis

Data were analyzed from June 1, 2019, to June 30, 2020. We used linear mixed-effects regression models to examine the association of antibiotic exposure with age and magnitude of BMI at peak and rebound, fitting separate regression models for each BMI milestone. For associations with age and magnitude of BMI at peak, we fit models using antibiotic exposure at 0 to 5 months of age only; this was the only exposure period that preceded the outcome. For associations with age and magnitude of BMI at rebound, we fit separate models using antibiotic exposure at 0 to 47 months of age and at specific age periods (0-5, 6-11, 12-23, 24-35, and 36-47 months of age), adjusting for age and magnitude of BMI at peak. For all analyses, we fit separate regression models for each type of antibiotic exposure (ie, any prescribed, broad-spectrum, and narrow-spectrum), categorized as yes vs no, at each of the age periods. We also assessed dose-response associations by separately examining each type of antibiotic exposure as a categorical count of increasing treatment episodes (0, 1, 2, 3, or ≥4) at each of these age periods.

In all models, we adjusted for child sex, race/ethnicity, preterm birth, asthma, infection episodes, complex chronic conditions, corticosteroid episodes, and number of clinical encounters at younger than 48 months. In models with age period–specific exposures, we adjusted for preceding antibiotic exposures and covariates contemporaneous with the exposure periods (eg, the model for antibiotic exposure at 12-23 months of age included antibiotic exposure at 0-5 and 6-11 months of age and corticosteroids, infections, and clinical encounters at 0-5, 6-11, and 12-23 months of age as covariates). We also accounted for clustering of children within the same network partner by including a random-effects term for each of the 26 network partners.

In sensitivity analyses, we excluded data from 5 network partners (3054 children) with the most atypical prescribing rates and the highest rates of likely errors in heights and weights (by comparing per-patient prescribing rates for study medications across network partners and with previously reported national estimates), to reduce misclassification of exposure and outcome. We also restricted all associations to children without complex chronic conditions (160 984 children). We performed all analyses as 2 sided using STATA, version 16.1 (StataCorp LP) and defined statistical significance at α = .05.

## Results

### Cohort Characteristics

Of the 183 444 children included in the study (mean age, 3.3 [range, 0-10.9] years; 95 228 [51.9%] were boys and 88 156 [48.1%] were girls; 80 043 [43.6%] were White individuals), 78.1% had at least 1 episode of any antibiotic exposure, 51.0% had at least 1 episode of broad-spectrum antibiotic exposure, and 65.0% had at least 1 episode of narrow-spectrum antibiotic exposure at 0 to 47 months of age. Compared with children with no exposure episodes, those with at least 4 episodes of any antibiotic exposure at 0 to 47 months of age were more likely to have an asthma diagnosis (21.8% vs 7.8%) and had more clinical encounters (97.0% vs 89.8%) and infections (96.2% vs 55.1%) (Table 1). The mean (SD) age at peak and rebound was 8.4 (2.1) months and 52.4 (18.9) months, respectively, and the mean (SD) magnitude of BMI at peak and rebound was 17.8 (1.5) and 15.7 (1.3), respectively. Girls (vs boys) had later mean (SD) age at peak (8.5 [2.2] vs 8.3 [2.0] months), earlier mean (SD) age at rebound (50.3 [19.1] vs 54.5 [18.5] months), and lower mean (SD) magnitude of BMI at both peak (17.6 [1.5] vs 18.1 [1.5]) and rebound (15.6 [1.3] vs 15.8 [1.3]) (eTable 1 in the Supplement).

**Table 1.** Characteristics of 183 444 Children With Estimable BMI Peak and Rebound According to Episodes of Any Antibiotic Exposure at 0 to 47 Months of Age

Characteristic	No. of episodes of any antibiotic exposure at 0-47 mo, No. (%) or % of patients^a^				
	0 (n = 45 628)	1 (n = 33 531)	2 (n = 25 662)	3 (n = 19 083)	≥4 (n = 59 540)
Child sex					
Male	22 861 (50.1)	17 067 (50.9)	13 349 (52.0)	9976 (52.3)	32 035 (53.8)
Female	22 767 (49.9)	16 464 (49.1)	12 313 (48.0)	9107 (47.7)	27 505 (46.2)
Race/ethnicity					
White, non-Hispanic	36.4	36.8	40.0	43.4	54.7
Black, non-Hispanic	29.6	33.2	32.0	30.0	22.5
Hispanic	22.4	18.1	16.3	15.0	12.3
Asian, non-Hispanic	4.0	4.4	4.0	3.6	2.9
Other, non-Hispanic^b^	3.5	3.7	3.5	3.6	3.3
Unknown	4.1	3.9	4.2	4.4	4.3
Preterm birth	6.7	7.0	7.1	7.0	7.0
Asthma	7.3	10.8	13.2	15.5	21.8
Complex chronic condition before 72 mo	13.2	11.1	10.9	11.0	13.1
Episodes of systemic corticosteroids at 0-47 mo, No.					
0	92.3	84.8	79.6	74.5	62.3
1	5.4	10.3	13.3	15.3	18.7
2	1.3	2.7	4.0	5.4	8.6
3	0.5	1.1	1.5	2.2	4.3
≥4	0.6	1.1	1.6	2.6	6.6
Episodes for presumed infectious illnesses at 0-47 mo, No.					
0	15.8	2.9	2.8	2.5	2.6
1	8.9	3.9	1.0	0.6	0.3
2	10.2	6.4	2.8	1.2	0.4
3	10.0	8.6	5.2	2.2	0.6
≥4	55.1	78.2	88.1	93.5	96.2
Clinical encounters at 0-47 mo, No.					
0	8.4	2.5	2.7	2.6	2.7
1	1.0	0.7	0.5	0.4	0.2
2	0.5	0.3	0.2	0.1	0.1
3	0.4	0.3	0.2	0.2	0.1
≥4	89.8	96.3	96.4	96.7	97.0

Abbreviation: BMI, body mass index.

^a^ Percentages have been rounded and may not total 100.

^b^ Includes Pacific Islander, American Indian, or Alaska Native.

### Associations With Age and Magnitude of BMI at Peak

**Figure 2.**
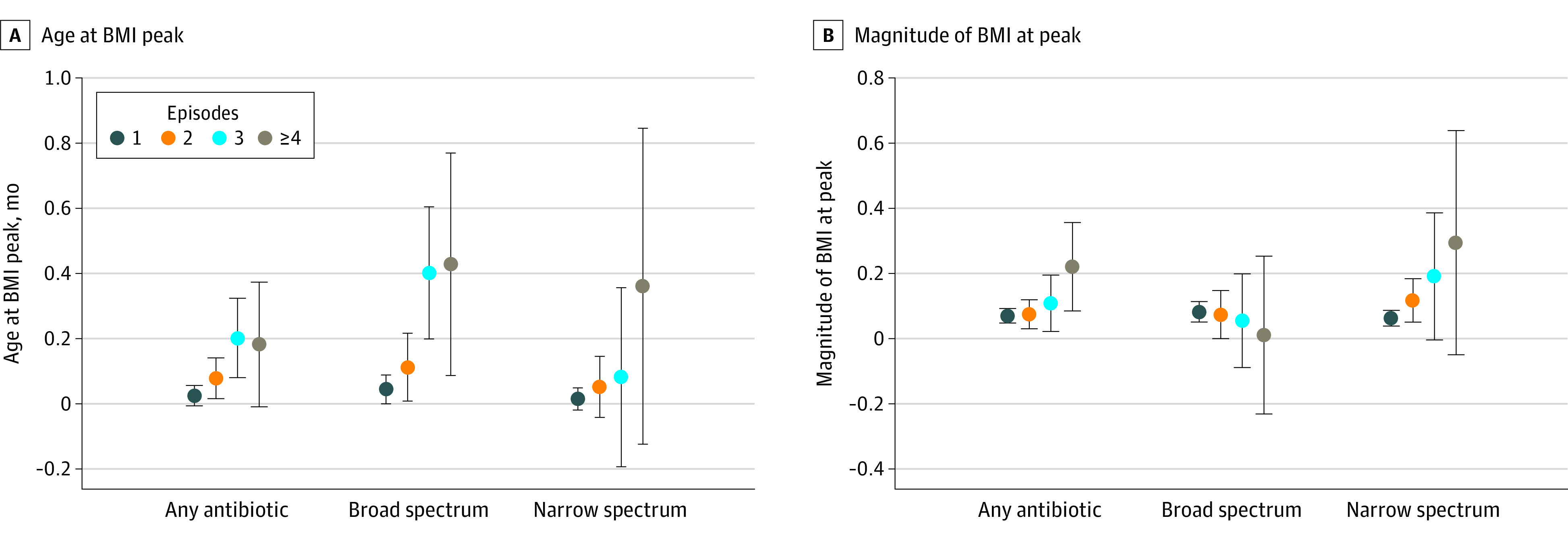
Associations of Antibiotic Exposure at 0 to 5 Months With Age and Magnitude of Body Mass Index (BMI) at Peak BMI is calculated as weight in kilograms divided by height in meters squared. Error bars indicate 95% CI.

Exposure to any antibiotics at 0 to 5 months of age (vs no exposure) was associated with later age (β coefficient, 0.05 months [95% CI, 0.02-0.08 months]) and higher BMI at peak (β coefficient, 0.09; [95% CI, 0.07-0.11]), with similar results for exposure to broad-spectrum antibiotics. Exposure to narrow-spectrum antibiotics at 0 to 5 months of age (vs no exposure) was associated with higher BMI at peak only (β coefficient, 0.08 [95% CI, 0.06 to 0.11]) (Table 2). Compared with no antibiotic exposure, receiving at least 4 antibiotic episodes of any type was associated with a 0.18-month later age at BMI peak (95% CI, −0.01 to 0.37 months); receiving at least 4 broad-spectrum antibiotic episodes was associated with a 0.43-month later age at peak (95% CI, 0.09-0.77 months), and receiving at least 4 narrow-spectrum antibiotic episodes was associated with a 0.36-month later age at peak (95% CI, −0.12 to 0.85 months) (Figure 2A). Magnitude of BMI peak was also higher with exposure to at least 4 antibiotic episodes of any type (0.22 [95% CI, 0.09-0.36]) and exposure to at least 4 narrow-spectrum antibiotic episodes (0.30 [95% CI, −0.05 to 0.64) compared with no antibiotic exposure (Figure 2B).

**Table 2.** Associations of Exposure to Antibiotics Before 48 Months With Age and Magnitude of BMI at Peak and Rebound by Timing of Antibiotic Exposure Periods

Exposure by age, mo	β Coefficient (95% CI)^a^			
	BMI peak^b^		BMI rebound	
	Age, mo	Magnitude	Age, mo	Magnitude
Any antibiotic				
0-5^c^	0.05 (0.02 to 0.08)	0.09 (0.07 to 0.11)	0.26 (0.01 to 0.51)	0.01 (0.00 to 0.03)
6-11^c^^,^^d^	NA	NA	0.00 (−0.20 to 0.20)	0.03 (0.02 to 0.04)
12-23^c^^,^^d^	NA	NA	−0.36 (−0.55 to −0.16)	0.03 (0.02 to 0.04)
24-35^c^^,^^d^	NA	NA	−0.63 (−0.83 to −0.43)	0.01 (0.00 to 0.02)
36-47^c^^,^^d^	NA	NA	−0.52 (−0.72 to −0.31)	0.01 (0.00 to 0.02)
0-47^c^	NA	NA	−0.60 (−0.81 to −0.39)	0.02 (0.01 to 0.03)
Broad-spectrum antibiotic				
0-5^c^	0.07 (0.03 to 0.01)	0.09 (0.07 to 0.12)	0.08 (−0.29 to 0.44)	0.01 (−0.00 to 0.03)
6-11^c^^,^^d^	NA	NA	0.24 (0.00 to 0.48)	0.02 (0.01 to 0.04)
12-23^c^^,^^d^	NA	NA	−0.25 (−0.46 to −0.05)	0.03 (0.02 to 0.04)
24-35^c^^,^^d^	NA	NA	−0.63 (−0.85 to −0.40)	0.02 (0.01 to 0.03)
36-47^c^^,^^d^	NA	NA	−0.64 (−0.88 to −0.40)	0.02 (0.01 to 0.03)
0-47^c^	NA	NA	−0.46 (−0.64 to −0.28)	0.03 (0.02 to 0.04)
Narrow-spectrum antibiotic				
0-5^c^	0.03 (–0.00 to 0.06)	0.08 (0.06 to 0.11)	0.28 (−0.01 to 0.56)	0.02 (0.00 to 0.03)
6-11^c^^,^^d^	NA	NA	−0.04 (−0.25 to 0.16)	0.03 (0.02 to 0.04)
12-23^c^^,^^d^	NA	NA	−0.36 (−0.55 to −0.16)	0.03 (0.02 to 0.03)
24-35^c^^,^^d^	NA	NA	−0.63 (−0.84 to −0.42)	0.01 (−0.00 to 0.02)
36-47^c^^,^^d^	NA	NA	−0.49 (−0.71 to −0.27)	0.01 (−0.00 to 0.02)
0-47^c^	NA	NA	−0.65 (−0.84 to −0.46)	0.03 (0.02 to 0.04)

Abbreviations: BMI, body mass index; NA, not applicable.

^a^ All associations were adjusted for child sex, race/ethnicity, preterm birth, asthma, infections, complex chronic conditions, corticosteroid exposure episodes, and clinical encounters.

^b^ Associations with age and magnitude of BMI at peak were only assessed for antibiotic exposure at 0 to 5 months of age.

^c^ Covariates contemporaneous with the exposure periods (corticosteroids, infections, and clinical encounters) were adjusted for age-specific antibiotic exposures.

^d^ Regression models were additionally adjusted for previous antibiotics exposure periods.

### Associations With Age and Magnitude of BMI at Rebound

**Figure 3.**
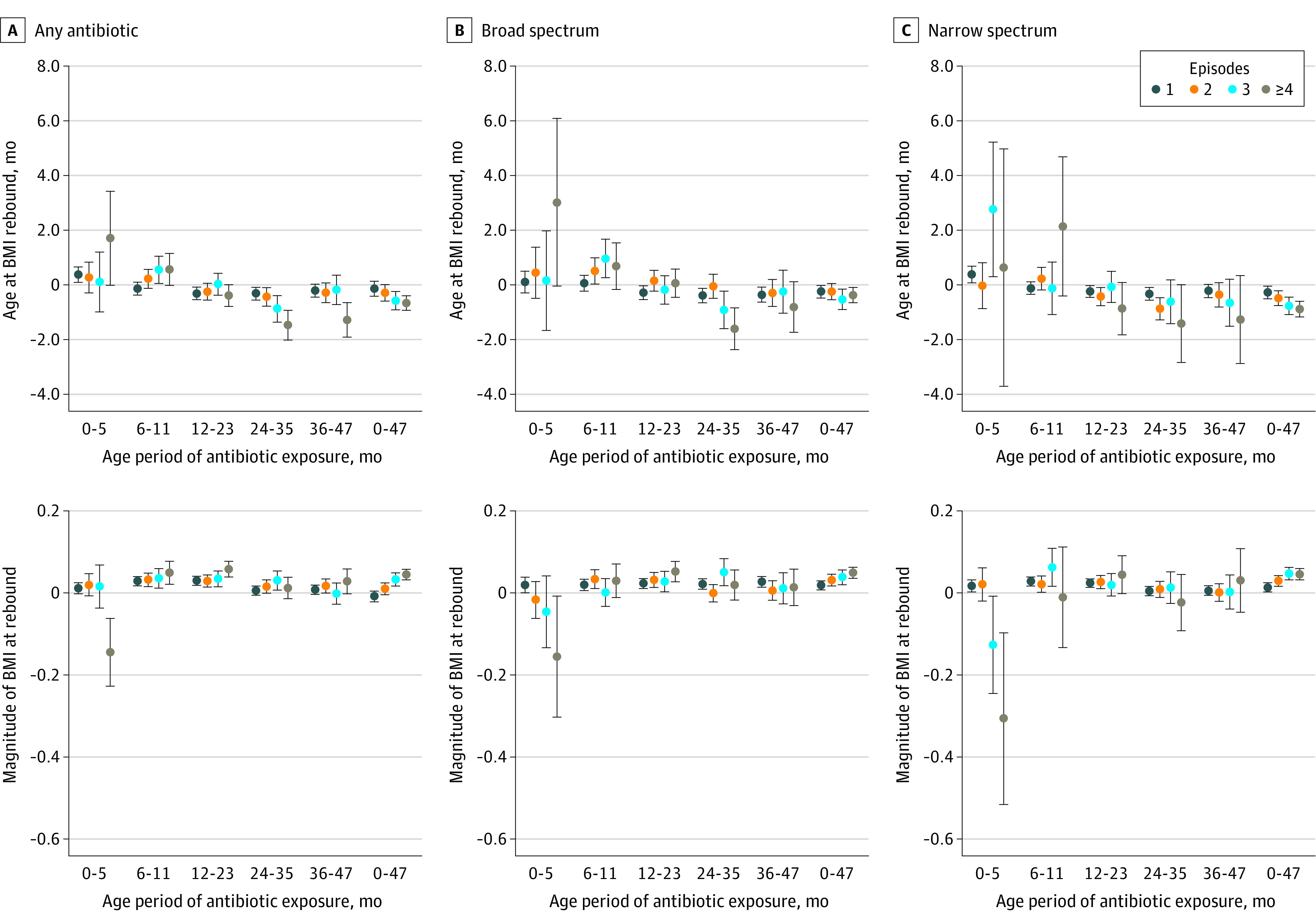
Associations of Antibiotic Exposure at 0 to 47 Months With Age and Magnitude of Body Mass Index (BMI) at Rebound Top row of graphs show association with age at BMI rebound; bottom row, magnitude of BMI at rebound. BMI is calculated as weight in kilograms divided by height in meters squared. Error bars indicate 95% CI.

Exposure to any antibiotics at 0 to 47 months of age (vs no exposure) was associated with an earlier age at BMI rebound (β coefficient, −0.60 months [95% CI, −0.81 to −0.39 months]) (Table 2), with evidence of a small dose-response association by −0.14 months for exposure to 1 episode, −0.29 months for exposure to 2 episodes, −0.58 months for exposure to 3 episodes, and −0.66 months for exposure to at least 4 episodes (Figure 3A, top). Effect estimates were slightly larger for those exposed to any vs no antibiotics at 24 to 35 months of age (−0.63 months [95% CI, −0.83 to −0.43 months]) and 36 to 47 months of age (−0.52 months [95% CI, −0.72 to −0.31 months]) than those exposed at 0 to 5 months of age (0.26 months [95% CI, 0.01-0.51 months]) or 6 to 11 months of age (0.00 months [95% CI, −0.20 to 0.20 months]). Compared with no antibiotic exposure, those with exposure to at least 4 antibiotic episodes of any type at 24 to 35 months of age (−1.47 months [95% CI, −2.02 to −0.93 months]) and 36 to 47 months of age (−1.28 months [95% CI, −1.91 to −0.65 months]) also had larger effect estimates for associations with age at rebound than those with exposure to at least 4 episodes at earlier periods (Figure 3A, top). Results were largely similar for exposure to broad- and narrow-spectrum antibiotics (Table 2 and Figure 3B and C, bottom).

Exposure to any antibiotics at 0 to 47 months of age (vs no exposure) was associated with higher BMI at rebound (0.02 [95% CI, 0.01-0.03]), with a dose-response association by −0.01 for exposure to 1 episode, 0.01 for exposure to 2 episodes, 0.03 for exposure to 3 episodes, and 0.04 for exposure to 4 or more episodes. Similar dose-response associations were observed for any antibiotic exposure at 6 to 11 (0.02 for exposure to 1 episode, 0.03 for exposure to 2 episodes, 0.04 for exposure to 3 episodes, and 0.05 for exposure to ≥4 episodes) and 12 to 23 (0.02 for exposure to 1 episode, 0.03 for exposure to 2 episodes, 0.03 for exposure to 3 episodes, and 0.06 for exposure to ≥4 episodes) months of age. Nevertheless, children with exposure to at least 4 antibiotic episodes of any type at 36 to 47 months of age (vs no exposure) had significantly higher BMI at rebound (0.03 [95% CI, 0.00-0.06]). However, children with exposure to at least 4 antibiotic episodes of any type at 0 to 5 months of age (vs no exposure) had lower BMI at rebound (−0.14 [95% CI, −0.22 to −0.06]) (Figure 3A, bottom). Dose-response associations with magnitude of BMI at rebound were also noted for exposure to broad- and narrow-spectrum antibiotics at 0 to 47 months of age. However, no consistent dose-response association was observed at other age periods for exposure to broad- and narrow-spectrum antibiotics (Figure 3B and C, bottom).

### Sensitivity Analyses

Our sensitivity analyses assessed associations of early-life antibiotic exposure with age and magnitude of BMI at peak and at rebound after excluding data from 5 network partners with the most atypical prescribing rates (eTable 2 and eFigures 2 and 3 in the Supplement) and among children without complex chronic conditions (eTables 3 to 5 in the Supplement). Patterns were similar to those observed in the full analytic cohort.

## Discussion

In this study, we used the PCORnet infrastructure to assemble a cohort of 183 444 children with antibiotic prescription data before 48 months of age and anthropometric data from birth through 131 months of age, making this the largest study to investigate the association between early childhood antibiotic exposure and longitudinal BMI trajectory milestone patterns. Overall, we identified small differences in age and magnitude of BMI at peak in infancy and rebound in early childhood associated with antibiotic exposure before 48 months of age. Effect estimates for associations with age at BMI rebound were larger for antibiotic exposure periods closer to the trajectory milestone. The type of antibiotics (broad or narrow spectrum) did not substantively affect the pattern of the observed associations.

Our findings are consistent with a recent analysis with the same cohort^5,9^ that found a positive association between BMI *z* score and obesity risk at 5 and 10 years of age after early-life antibiotic exposure. Children who received antibiotics before 24 months of age had small mean (SD) increases in BMI *z* scores at 5 (0.04) and 10 (0.03) years of age and a small risk of overweight and obesity at 5 (odds ratio, 1.05 [95% CI, 1.03-1.07]) and 10 (odds ratio, 1.02 [95% CI, 0.97-1.07]) years of age.^5,9^ The small effect estimates observed in our study also closely align with previous studies of association of early exposure to antibiotics with childhood growth trajectory.^4,16^ Gerber et al^4^ reported a 2% increase in the growth rate for children who were exposed to antibiotics in the first 2 years, whereas Schwartz et al^16^ estimated excess weight gains of 0.73 to 1.50 kg from 3 to 18 years of age for children who received antibiotics. The similarity in the magnitude of associations across these different studies adds credibility to our findings.

Past studies have shown that exposure to antibiotics at early infancy (<6 months of age) were associated with increases in BMI and risk of obesity at 10 to 36 months of age,^14,31^ but not at later ages.^4,32^ Our present study supports these observations by showing stronger associations with magnitude of BMI at peak (approximately 8 months of age) than at rebound (approximately 52 months of age) after antibiotic exposure at 0 to 5 months of age. Other studies have documented longer-term effects on overweight and obesity in late childhood after exposure to antibiotics at early infancy.^15,33,34^ These studies, however, varied considerably on adjustments made in their analyses, which may have explained the differences. In addition, our observations of dose-response associations for repeated antibiotic exposure with magnitude of BMI peak and rebound is consistent with previous studies showing similar associations with BMI *z* scores or risk of obesity.^3,13,35^ Moreover, the dose-response association with magnitude of BMI rebound seemed apparent only at 6 to 11 and 12 to 23 months of age. It has been proposed that both dosage and timing of antibiotic regimens during early life may have qualitatively different effects on post–antibiotic treatment growth.^36^ Further longitudinal studies are clearly warranted.

Our study contributes to the extant literature by demonstrating associations of antibiotic exposure after 24 months with age and magnitude of BMI rebound in childhood. Effect estimates for associations with age at BMI rebound were also larger for antibiotic exposure periods closer to the trajectory milestone. Although we have no clear biological explanation for these observations, these findings are consistent with prior studies^37,38,39^ that have shown stronger associations with health outcomes for exposures at later ages than at earlier ages. Taken together, our results suggest a short-term effect of antibiotic exposure on weight in infancy and childhood; this effect may not be sustained over the long term.

Our results showed weak associations overall with age at BMI peak and rebound and magnitude of BMI at peak and rebound after antibiotic exposure before 48 months of age. Even for exposure to 4 or more antibiotic episodes, the associations with age at BMI peak and rebound and the magnitude of BMI at peak and rebound were relatively small. To put these findings in perspective, a recent study from a prebirth cohort in Boston^17^ showed that children born to mothers with obesity (vs those born to mothers without obesity) had an age at rebound that was earlier by approximately 11 months and magnitude of BMI rebound that was higher by 0.6. Later age and greater magnitude at BMI peak and an earlier age at BMI rebound is significantly associated with obesity and cardiometabolic risk in later childhood.^18,19^ Our findings, however, suggest that the small changes to these BMI trajectory milestones after antibiotic exposure would have minimal clinical importance, if any. Thus, although excessive antibiotic exposure during early life remains a valid concern, the small associations with BMI trajectory patterns after antibiotic exposure should not affect the individual decision to prescribe antibiotics.

### Strengths and Limitations

Strengths of our study include its large analytic sample and multiple measures of growth throughout childhood. This enabled us to model complete BMI trajectories from birth until late childhood instead of being restricted to segments of trajectories (eg, from birth to early childhood^40^ or from early to late childhood^41^) commonly seen in other studies. One limitation, however, is that we captured antibiotic prescribing data electronically and not based on pharmacy dispensing or claims, which might have led to misclassification of the exposure. However, previous analyses among a subset of participating institutions from PCORnet^24^ showed the prescribing data had good sensitivity in identifying patients who filled the antibiotic prescription. Further, we accounted for mostly oral antibiotic prescriptions, although we did capture intramuscular ceftriaxone and penicillin use. The lack of additional intravenous antibiotics may have biased the results to the null. Second, we estimated BMI peak and rebound from statistical models, rather than direct observations using visual inspection of individual BMI-for-age curves. However, our models showed mean residual errors (differences between observed and predicted BMI) that were close to zero across all ages. Furthermore, modeling the entire curve from birth, as we did, is likely to provide more precise estimations of peak and rebound than visual inspection of raw BMI values, which is subject to large interobserver variation.^25,27^ Third, we did not have data on some confounders that could potentially be associated with antibiotic exposure and BMI trajectory patterns, including socioeconomic status, diet, and breastfeeding status. Electronic health records rarely contain structured data for these variables. Fourth, the overrepresentation of urban environments with large health care systems in our analytic sample might limit the generalizability of our findings.

## Conclusions

The findings of this cohort study showed small associations between antibiotic exposure before 48 months of age and BMI trajectory milestones that are known risk markers of later obesity (ie, later age at BMI peak, earlier age at BMI rebound, and higher BMI at each milestone). Considering these findings, the small risk of an altered BMI trajectory milestone pattern associated with antibiotic exposure is unlikely to be a key factor in any individual prescribing decision for children.

## Notes

Supplement. **eTable 1.** Age and Magnitude of BMI at Peak and Rebound According to Demographic and Clinical Characteristics (n = 183 444) **eTable 2.** Associations of Exposure to Antibiotics at Less Than 48 Months With Age and Magnitude of BMI at Peak and Rebound, by Timing of Antibiotic Exposure Periods, After Excluding Data From 5 Network Partners With the Most Atypical Prescribing Rates (n = 180 390) **eTable 3.** Associations of Antibiotic Exposure at 0-5 Months With Age and Magnitude of BMI at Peak, by Complex Chronic Condition Status **eTable 4.** Associations of Antibiotic Exposure at 0-47 Months With Age at BMI Rebound, by Complex Chronic Condition Status **eTable 5.** Associations of Antibiotic Exposure at 0-47 Months With Magnitude of BMI Rebound, by Complex Chronic Condition Status **eFigure 1.** Scatterplot of Standardized Residuals **eFigure 2.** Associations of Antibiotic Exposure at 0-5 Months With Age and Magnitude of BMI at Peak, After Excluding Data From 5 Network Partners With the Most Atypical Prescribing Rates **eFigure 3.** Associations of Antibiotic Exposure at 0-47 Months With Age and Magnitude of BMI at Rebound, After Excluding Data From 5 Network Partners With the Most Atypical Prescribing Rates
