# Supplementary material for: Association of Early Antibiotic Exposure With Childhood Body Mass Index Trajectory Milestones
Source: JAMA Netw Open. 2021 Jul 12;4(7):e2116581. doi: 10.1001/jamanetworkopen.2021.16581 (PMC8276083; doi:10.1001/jamanetworkopen.2021.16581)
Supplement: Supplement. — eTable 1. Age and Magnitude of BMI at Peak and Rebound According to Demographic and Clinical Characteristics (n = 183 444) eTable 2. Associations of Exposure to Antibiotics at Less Than 48 Months With Age and Magnitude of BMI at Peak and Rebound, by Timing of Antibiotic Exposure Periods, After Excluding Data From 5 Network Partners With the Most Atypical Prescribing Rates (n = 180 390) eTable 3. Associations of Antibiotic Exposure at 0-5 Months With Age and Magnitude of BMI at Peak, by Complex Chronic Condition Status eTable 4. Associations of Antibiotic Exposure at 0-47 Months With Age at BMI Rebound, by Complex Chronic Condition Status eTable 5. Associations of Antibiotic Exposure at 0-47 Months With Magnitude of BMI Rebound, by Complex Chronic Condition Status eFigure 1. Scatterplot of Standardized Residuals eFigure 2. Associations of Antibiotic Exposure at 0-5 Months With Age and Magnitude of BMI at Peak, After Excluding Data From 5 Network Partners With the Most Atypical Prescribing Rates eFigure 3. Associations of Antibiotic Exposure at 0-47 Months With Age and Magnitude of BMI at Rebound, After Excluding Data From 5 Network Partners With the Most Atypical Prescribing Rates [file jamanetwopen-e2116581-s001.pdf]

Supplementary Online Content

Aris IM, Lin PID, Rifas-Shiman SL, et al. Association of early antibiotic exposure with childhood body mass index trajectory milestones. *JAMA Netw Open*. 2021;4(7):e2116581. doi:10.1001/jamanetworkopen.2021.16581

eTable 1. Age and Magnitude of BMI at Peak and Rebound According to Demographic and Clinical Characteristics (n = 183 444)

eTable 2. Associations of Exposure to Antibiotics at Less Than 48 Months With Age and Magnitude of BMI at Peak and Rebound, by Timing of Antibiotic Exposure Periods, After Excluding Data From 5 Network Partners With the Most Atypical Prescribing Rates (n = 180 390)

eTable 3. Associations of Antibiotic Exposure at 0-5 Months With Age and Magnitude of BMI at Peak, by Complex Chronic Condition Status

eTable 4. Associations of Antibiotic Exposure at 0-47 Months With Age at BMI Rebound, by Complex Chronic Condition Status

eTable 5. Associations of Antibiotic Exposure at 0-47 Months With Magnitude of BMI Rebound, by Complex Chronic Condition Status

eFigure 1. Scatterplot of Standardized Residuals

eFigure 2. Associations of Antibiotic Exposure at 0-5 Months With Age and Magnitude of BMI at Peak, After Excluding Data From 5 Network Partners With the Most Atypical Prescribing Rates

eFigure 3. Associations of Antibiotic Exposure at 0-47 Months With Age and Magnitude of BMI at Rebound, After Excluding Data From 5 Network Partners With the Most Atypical Prescribing Rates

This supplementary material has been provided by the authors to give readers additional information about their work.

© 2021 Aris IM et al. *JAMA Network Open*.

eTable 1. Age and Magnitude of BMI at Peak and Rebound According to Demographic and Clinical Characteristics (n = 183 444)

		BMI peak		BMI rebound	
		Age (months)	Magnitude (kg/m^2)	Age (months)	Magnitude (kg/m^2)
		Mean (SD)			
**Child sex**					
	Male	8.3 (2.0)	18.1 (1.5)	54.5 (18.5)	15.8 (1.3)
	Female	8.5 (2.2)	17.6 (1.5)	50.3 (19.1)	15.6 (1.3)
**Race/ethnicity**					
	White, non-Hispanic	8.5 (2.2)	17.7 (1.5)	54.0 (18.7)	15.6 (1.2)
	Black, non-Hispanic	8.2 (1.9)	18.0 (1.6)	51.7 (18.5)	15.8 (1.3)
	Hispanic	8.4 (2.1)	17.9 (1.6)	49.3 (19.7)	15.9 (1.4)
	Asian, non-Hispanic	8.1 (2.0)	17.5 (1.5)	53.9 (19.5)	15.3 (1.2)
	Other, non-Hispanic	8.4 (2.1)	17.8 (1.6)	52.4 (18.8)	15.7 (1.3)
	Unknown	8.3 (2.0)	17.7 (1.5)	53.3 (19.0)	15.7 (1.3)
**Preterm**					
	No	8.3 (2.1)	17.8 (1.5)	52.4 (18.9)	15.7 (1.3)
	Yes	8.9 (2.4)	17.4 (1.6)	53.6 (18.8)	15.4 (1.3)
**Asthma**					
	No	8.4 (2.1)	17.8 (1.5)	52.5 (18.9)	15.7 (1.3)
	Yes	8.4 (2.1)	18.2 (1.6)	52.3 (18.9)	16.0 (1.4)
**Complex chronic condition <72 months**					
	No	8.2 (2.0)	17.8 (1.5)	52.3 (18.9)	15.7 (1.3)
	Yes	8.9 (2.7)	17.8 (1.6)	53.4 (18.9)	15.6 (1.4)
**Systemic corticosteroids episodes 0-47 months**					
	0	8.4 (2.1)	17.7 (1.5)	52.3 (19.0)	15.7 (1.3)
	1	8.3 (2.0)	18.0 (1.5)	52.9 (18.7)	15.8 (1.3)
	2	8.3 (2.0)	18.2 (1.6)	53.3 (18.5)	15.9 (1.3)
	3	8.4 (2.2)	18.2 (1.6)	53.0 (18.5)	16.0 (1.4)
	≥4	8.5 (2.3)	18.4 (1.7)	52.1 (18.7)	16.1 (1.4)
**Episodes for presumed infectious illnesses 0-47 months**					
	0	8.5 (2.2)	17.7 (1.5)	52.1 (18.6)	15.8 (1.3)
	1	8.3 (2.1)	17.7 (1.5)	53.2 (18.5)	15.7 (1.3)
	2	8.3 (2.1)	17.7 (1.5)	52.9 (18.9)	15.6 (1.3)
	3	8.3 (2.0)	17.7 (1.5)	52.9 (18.6)	15.6 (1.3)
	≥4	8.4 (2.1)	17.8 (1.5)	52.4 (19.0)	15.7 (1.3)
**Number of clinical encounters 0-47 months**					
	0	8.5 (2.3)	17.8 (1.5)	51.4 (18.5)	15.8 (1.3)
	1	8.4 (1.8)	17.9 (1.5)	51.1 (18.8)	15.7 (1.4)
	2	8.3 (1.9)	17.7 (1.6)	51.9 (18.9)	15.6 (1.4)
	3	8.5 (2.3)	17.4 (1.5)	50.8 (18.7)	15.4 (1.3)
	≥4	8.4 (2.1)	17.8 (1.5)	52.5 (18.9)	15.7 (1.3)

© 2021 Aris IM et al. *JAMA Network Open*.

eTable 2. Associations of Exposure to Antibiotics at Less Than 48 Months With Age and Magnitude of BMI at Peak and Rebound, by Timing of Antibiotic Exposure Periods, After Excluding Data From 5 Network Partners With the Most Atypical Prescribing Rates (n = 180 390)

	**BMI peak**		**BMI rebound**	
	**Age (months)**	**Magnitude (kg/m^2)**	**Age (months)**	**Magnitude (kg/m^2)**
	**β (95% CI)[a]**			
Any, mo				
0–5[b]	0.04 (0.02, 0.07)	0.09 (0.07, 0.11)	0.28 (0.03, 0.59)	0.02 (0.00, 0.03)
6–11[b,c]	–[d]	–	0.00 (-0.20, 0.20)	0.03 (0.02, 0.04)
12–23[b,c]	–	–	-0.34 (-0.54, -0.19)	0.03 (0.02, 0.04)
24–35[b,c]	–	–	-0.62 (-0.82, -0.42)	0.01 (0.00, 0.02)
36–47[b,c]	–	–	-0.54 (-0.75, -0.33)	0.01 (0.00, 0.02)
0–47[b]	–	–	-0.59 (-0.80, -0.37)	0.02 (0.01, 0.03)
Broad-spectrum, mo				
0–5[b]	0.06 (0.02, 0.10)	0.10 (0.07, 0.12)	0.12 (-0.25, 0.49)	0.02 (-0.01, 0.03)
6–11[b,c]	–	–	0.23 (-0.01, 0.47)	0.03 (0.01, 0.04)
12–23[b,c]	–	–	-0.26 (-0.47, -0.05)	0.03 (0.02, 0.04)
24–35[b,c]	–	–	-0.60 (-0.83, -0.37)	0.02 (0.01, 0.03)
36–47[b,c]	–	–	-0.65 (-0.89, -0.41)	0.02 (0.01, 0.03)
0–47[b]	–	–	-0.46 (-0.64, -0.28)	0.03 (0.02, 0.04)
Narrow-spectrum, mo				
0–5[b]	0.03 (0.00, 0.06)	0.08 (0.06, 0.10)	0.29 (0.00, 0.58)	0.02 (0.00, 0.03)
6–11[b,c]	–	–	-0.05 (-0.26, 0.15)	0.03 (0.02, 0.04)
12–23[b,c]	–	–	-0.34 (-0.53, -0.15)	0.02 (0.01, 0.03)
24–35[b,c]	–	–	-0.64 (-0.85, -0.43)	0.01 (0.00, 0.02)
36–47[b,c]	–	–	-0.50 (-0.72, -0.28)	0.01 (0.00, 0.02)
0–47[b]	–	–	-0.64 (-0.83, -0.45)	0.03 (0.02, 0.04)

[a] All associations were adjusted for child sex, race/ethnicity, preterm birth, asthma, infections, complex chronic conditions, corticosteroid episodes and clinical encounters.
[b] Covariates contemporaneous with the exposure periods (corticosteroids, infections and clinical encounters) were adjusted for age-specific antibiotic exposures.
[c] Additionally adjusted for previous antibiotics exposure periods.
[d] Associations with age and magnitude of BMI at peak were only assessed for antibiotic exposure at 0–5 months.

© 2021 Aris IM et al. *JAMA Network Open*.

eTable 3. Associations of Antibiotic Exposure at 0-5 Months With Age and Magnitude of BMI at Peak, by Complex Chronic Condition Status

	Without complex chronic conditions (n=160,984)			With complex chronic conditions (n=22,460)		
	**Any antibiotic**	**Broad-spectrum**	**Narrow-spectrum**	**Any antibiotic**	**Broad-spectrum**	**Narrow-spectrum**
		**β (95% CI)[a]**			**β (95% CI)[a]**	
Age of BMI at peak						
No exposure	ref	ref	ref	ref	ref	ref
1 episode	0.04	0.02	0.06	-0.02	0.11	-0.22
	(0.01,0.07)	(-0.02,0.07)	(0.02,0.09)	(-0.13,0.08)	(-0.02,0.23)	(-0.35,-0.10)
2 episodes	0.1	0.14	0.15	-0.01	0.05	-0.30
	(0.04,0.17)	(0.01,0.26)	(0.05,0.25)	(-0.18,0.16)	(-0.18,0.27)	(-0.57,-0.03)
3 episodes	0.21	0.08	-0.15	0.14	0.64	0.30
	(0.06,0.35)	(-0.20,0.36)	(-0.51,0.22)	(-0.13,0.41)	(0.27,1.01)	(-0.22,0.82)
≥4 episodes	0.04	0.15	-0.3	0.26	0.5	0.43
	(-0.22,0.31)	(-0.45,0.76)	(-1.40,0.81)	(-0.10,0.61)	(-0.04,1.04)	(-0.27,1.14)
Magnitude of BMI at peak						
No exposure	ref	ref	ref	ref	ref	ref
1 episode	0.05	0.08	0.04	0.15	0.1	0.17
	(0.03,0.08)	(0.04,0.11)	(0.02,0.07)	(0.09,0.21)	(0.03,0.18)	(0.10,0.24)
2 episodes	0.06	0.03	0.09	0.13	0.17	0.20
	(0.01,0.11)	(-0.07,0.12)	(0.02,0.17)	(0.03,0.23)	(0.04,0.30)	(0.05,0.36)
3 episodes	0.11	0.28	0.12	0.14	-0.09	0.29
	(0.01,0.22)	(0.07,0.49)	(-0.15,0.39)	(-0.02,0.29)	(-0.31,0.12)	(-0.01,0.59)
≥4 episodes	0.24	-0.15	0.09	0.25	0.14	0.38
	(0.05,0.44)	(-0.60,0.30)	(-0.73,0.91)	(0.04,0.45)	(-0.17,0.46)	(-0.02,0.79)

[a] Adjusted for child sex, race/ethnicity, preterm birth, asthma, infections, corticosteroid episodes and clinical encounters at 0–5 months of age.

© 2021 Aris IM et al. *JAMA Network Open*.

eTable 4. Associations of Antibiotic Exposure at 0-47 Months With Age at BMI Rebound, by Complex Chronic Condition Status

	**Without complex chronic conditions (n=160,984)[a]**						**With complex chronic conditions (n=22,460)[a]**					
	**0–5 mo[b]**	**6–11 mo[b,c]**	**12–23 mo[b,c]**	**24–35 mo[b,c]**	**36–47 mo[b,c]**	**0–47 mo[b]**	**0–5 mo[b]**	**6–11 mo[b,c]**	**12–23 mo[b,c]**	**24–35 mo[b,c]**	**36–47 mo[b,c]**	**0–47 mo[b]**
Any												
No exposure	ref	ref	ref	ref	ref	ref	ref	ref	ref	ref	ref	ref
1 episode	0.23	-0.19	-0.31	-0.34	-0.22	-0.13	1.04	0.08	-0.45	-0.28	-0.25	-0.31
	(-0.08,0.53)	(-0.44,0.06)	(-0.56,-0.07)	(-0.59,-0.10)	(-0.47,0.03)	(-0.42,0.16)	(0.31,1.76)	(-0.59,0.75)	(-1.12,0.22)	(-0.96,0.40)	(-0.94,0.44)	(-1.11,0.48)
2 episodes	0.17	0.15	-0.5	-0.56	-0.38	-0.27	0.59	0.51	1.22	0.12	0.00	-0.60
	(-0.47,0.81)	(-0.22,0.52)	(-0.82,-0.17)	(-0.93,-0.20)	(-0.77,0.00)	(-0.59,0.05)	(-0.59,1.76)	(-0.44,1.47)	(0.35,2.09)	(-0.83,1.06)	(-1.01,1.00)	(-1.48,0.27)
3 episodes	0.06	0.6	-0.1	-1.05	-0.57	-0.52	0.28	-0.01	0.34	-0.29	1.46	-1.21
	(-1.31,1.43)	(0.06,1.13)	(-0.52,0.33)	(-1.57,-0.53)	(-1.15,0.01)	(-0.88,-0.17)	(-1.55,2.12)	(-1.39,1.37)	(-0.79,1.47)	(-1.60,1.02)	(0.03,2.89)	(-2.18,-0.23)
≥4 episodes	1.77	0.38	-0.59	-1.69	-1.5	-0.81	1.69	1.56	0.49	-0.83	-0.75	0.07
	(-0.72,4.26)	(-0.25,1.01)	(-1.02,-0.16)	(-2.29,-1.09)	(-2.20,-0.79)	(-1.09,-0.52)	(-0.73,4.11)	(-0.04,3.16)	(-0.59,1.57)	(-2.14,0.49)	(-2.20,0.70)	(-0.65,0.79)
Broad-spectrum												
No exposure	ref	ref	ref	ref	ref	ref	ref	ref	ref	ref	ref	ref
1 episode	0.09	0.09	-0.34	-0.29	-0.40	-0.29	0.15	-0.2	-0.02	-1.13	-0.16	0.03
	(-0.35,0.54)	(-0.22,0.40)	(-0.61,-0.07)	(-0.57,-0.01)	(-0.69,-0.10)	(-0.54,-0.05)	(-0.72,1.02)	(-0.97,0.57)	(-0.73,0.70)	(-1.88,-0.37)	(-0.94,0.62)	(-0.66,0.72)
2 episodes	0.26	0.45	0	-0.16	-0.32	-0.24	0.88	0.70	1.03	0.46	-0.22	-0.32
	(-0.92,1.44)	(-0.07,0.97)	(-0.41,0.41)	(-0.63,0.32)	(-0.86,0.21)	(-0.56,0.07)	(-0.67,2.43)	(-0.51,1.90)	(0.01,2.04)	(-0.71,1.63)	(-1.49,1.05)	(-1.17,0.53)
3 episodes	1.34	0.91	-0.13	-0.91	-0.29	-0.58	-0.79	1.08	-0.70	-1.13	-0.19	-0.16
	(-1.33,4.01)	(0.15,1.68)	(-0.69,0.43)	(-1.66,-0.16)	(-1.16,0.58)	(-0.99,-0.18)	(-3.32,1.73)	(-0.73,2.90)	(-2.12,0.72)	(-2.82,0.56)	(-2.04,1.66)	(-1.20,0.89)
≥4 episodes	4.22	0.39	-0.02	-1.45	-0.90	-0.38	2.53	2.28	0.35	-2.14	-0.18	-0.30
	(-1.53,9.96)	(-0.54,1.31)	(-0.58,0.54)	(-2.32,-0.59)	(-1.96,0.17)	(-0.69,-0.08)	(-1.18,6.23)	(0.06,4.49)	(-0.99,1.69)	(-3.79,-0.48)	(-2.08,1.72)	(-1.03,0.44)
Narrow-spectrum												
No exposure	ref	ref	ref	ref	ref	ref	ref	ref	ref	ref	ref	ref
1 episode	0.31	-0.17	-0.31	-0.42	-0.24	-0.27	0.59	0.17	0.14	0.26	-0.29	-0.39
	(-0.01,0.64)	(-0.41,0.08)	(-0.54,-0.08)	(-0.67,-0.18)	(-0.49,0.02)	(-0.52,-0.02)	(-0.25,1.43)	(-0.52,0.87)	(-0.51,0.79)	(-0.44,0.97)	(-1.02,0.44)	(-1.06,0.27)
2 episodes	-0.12	0.2	-0.62	-1.01	-0.50	-0.40	0.13	0.27	0.85	-0.25	0.10	-1.48
	(-1.07,0.83)	(-0.24,0.64)	(-0.98,-0.27)	(-1.45,-0.58)	(-0.98,-0.03)	(-0.69,-0.11)	(-1.70,1.95)	(-1.00,1.54)	(-0.17,1.87)	(-1.44,0.93)	(-1.20,1.40)	(-2.28,-0.68)
3 episodes	1.99	-0.19	-0.39	-1.05	-1.00	-0.75	3.46	0.01	1.91	1.85	0.61	-1.28
	(-1.44,5.43)	(-1.22,0.84)	(-1.00,0.21)	(-1.91,-0.19)	(-1.94,-0.07)	(-1.09,-0.41)	(-0.09,7.02)	(-2.79,2.80)	(0.26,3.56)	(-0.38,4.08)	(-1.64,2.86)	(-2.24,-0.31)
≥4 episodes	-1.29	2.5	-0.87	-1.85	-2.32	-1.13	0.84	1.11	-1.24	-0.48	1.51	0.52
	(-11.77,9.19)	(-0.44,5.43)	(-1.91,0.17)	(-3.46,-0.24)	(-4.15,-0.49)	(-1.44,-0.82)	(-3.98,5.65)	(-4.09,6.30)	(-3.77,1.29)	(-3.61,2.64)	(-1.95,4.97)	(-0.27,1.31)

[a] All associations were adjusted for child sex, race/ethnicity, preterm birth, asthma, infections, corticosteroid episodes and clinical encounters.
[b] Covariates contemporaneous with the exposure periods (corticosteroids, infections and clinical encounters) were adjusted for age-specific antibiotic exposures.
[c] Additionally adjusted for previous antibiotics exposure periods.

© 2021 Aris IM et al. *JAMA Network Open*.

eTable 5. Associations of Antibiotic Exposure at 0-47 Months With Magnitude of BMI Rebound, by Complex Chronic Condition Status

	**Without complex chronic conditions (n=160,984)[a]**						**With complex chronic conditions (n=22,460)[a]**					
	**0–5 mo[b]**	**6–11 mo[b,c]**	**12–23 mo[b]**	**24–35 mo[b,c]**	**36–47 mo[b]**	**0–47 mo[b,c]**	**0–5 mo[b]**	**6–11 mo[b,c]**	**12–23 mo[b]**	**24–35 mo[b,c]**	**36–47 mo[b]**	**0–47 mo[b,c]**
Any												
No exposure	ref	ref	ref	ref	ref	ref	ref	ref	Ref	ref	ref	ref
1 episode	0.02	0.03	0.03	0.01	0.01	-0.01	-0.03	0.01	-0.02	0.00	0.03	-0.01
	(0.00,0.03)	(0.02,0.04)	(0.02,0.04)	(-0.00,0.02)	(-0.01,0.02)	(-0.02,0.01)	(-0.06,0.01)	(-0.02,0.04)	(-0.09,0.05)	(-0.04,0.03)	(-0.01,0.06)	(-0.05,0.03)
2 episodes	0.02	0.03	0.04	0.02	0.02	0.01	0	0.01	0	0.00	0.02	0.02
	(-0.01,0.05)	(0.02,0.05)	(0.02,0.05)	(0.00,0.04)	(-0.00,0.04)	(-0.01,0.03)	(-0.06,0.06)	(-0.03,0.06)	(-0.08,0.09)	(-0.05,0.04)	(-0.03,0.07)	(-0.03,0.06)
3 episodes	0.03	0.03	0.04	0.03	0.02	0.03	-0.02	0.04	-0.01	0.05	-0.09	0.05
	(-0.03,0.10)	(0.01,0.06)	(0.02,0.06)	(0.00,0.05)	(-0.01,0.04)	(0.02,0.05)	(-0.11,0.08)	(-0.03,0.11)	(-0.12,0.10)	(-0.01,0.12)	(-0.16,-0.02)	(-0.00,0.10)
≥4 episodes	-0.1	0.06	0.06	0.02	0.04	0.05	-0.19	-0.03	-0.02	-0.01	0.00	0.00
	(-0.22,0.02)	(0.03,0.09)	(0.04,0.08)	(-0.01,0.05)	(0.01,0.07)	(0.04,0.07)	(-0.31,-0.07)	(-0.11,0.05)	(-0.14,0.09)	(-0.08,0.06)	(-0.07,0.07)	(-0.04,0.03)
Broad-spectrum												
No exposure	ref	ref	ref	ref	ref	ref	ref	ref	ref	ref	ref	ref
1 episode	0.02	0.02	0.02	0.02	0.03	0.02	0.03	0.01	0.01	0.03	0.01	-0.01
	(-0.00,0.04)	(0.01,0.04)	(0.01,0.04)	(0.01,0.03)	(0.02,0.04)	(0.01,0.03)	(-0.01,0.08)	(-0.03,0.05)	(-0.02,0.05)	(-0.00,0.07)	(-0.03,0.05)	(-0.04,0.03)
2 episodes	-0.01	0.04	0.04	0.00	0.00	0.03	-0.02	0.01	-0.01	0.02	0.02	0.02
	(-0.07,0.04)	(0.01,0.06)	(0.02,0.06)	(-0.03,0.02)	(-0.02,0.03)	(0.02,0.05)	(-0.10,0.06)	(-0.05,0.07)	(-0.06,0.04)	(-0.04,0.08)	(-0.04,0.09)	(-0.02,0.06)
3 episodes	-0.06	0.01	0.02	0.05	0.03	0.04	-0.02	-0.07	0.06	0.07	-0.08	0.01
	(-0.19,0.07)	(-0.02,0.05)	(-0.00,0.05)	(0.01,0.08)	(-0.01,0.07)	(0.02,0.06)	(-0.15,0.11)	(-0.16,0.02)	(-0.02,0.13)	(-0.01,0.16)	(-0.17,0.02)	(-0.04,0.06)
≥4 episodes	-0.14	0.05	0.05	0.03	0.03	0.06	-0.15	-0.08	0.05	0.01	-0.04	0.01
	(-0.42,0.13)	(0.00,0.09)	(0.03,0.08)	(-0.01,0.07)	(-0.02,0.08)	(0.04,0.07)	(-0.34,0.04)	(-0.19,0.04)	(-0.02,0.12)	(-0.08,0.09)	(-0.13,0.06)	(-0.03,0.05)
Narrow-spectrum												
No exposure	ref	ref	ref	ref	ref	ref	ref	ref	ref	ref	ref	ref
1 episode	0.02	0.03	0.03	0.01	0.01	0.01	-0.02	0.02	0.01	0.00	0.01	0.04
	(0.01,0.04)	(0.02,0.04)	(0.02,0.04)	(-0.01,0.02)	(-0.01,0.02)	(-0.00,0.02)	(-0.07,0.02)	(-0.02,0.05)	(-0.02,0.04)	(-0.04,0.03)	(-0.03,0.05)	(0.00,0.07)
2 episodes	0.03	0.02	0.03	0.01	0.00	0.03	-0.02	0.04	-0.01	0.00	0.03	0.07
	(-0.02,0.08)	(-0.00,0.04)	(0.01,0.05)	(-0.01,0.03)	(-0.02,0.02)	(0.01,0.04)	(-0.11,0.08)	(-0.03,0.10)	(-0.06,0.04)	(-0.06,0.06)	(-0.04,0.09)	(0.02,0.11)
3 episodes	-0.1	0.06	0.03	0.02	0.01	0.05	-0.16	0.04	-0.08	-0.06	-0.04	0.06
	(-0.26,0.07)	(0.01,0.11)	(0.00,0.06)	(-0.02,0.06)	(-0.03,0.06)	(0.03,0.06)	(-0.34,0.02)	(-0.10,0.19)	(-0.17,0.00)	(-0.17,0.05)	(-0.16,0.07)	(0.01,0.11)
≥4 episodes	-0.24	-0.01	0.04	0.01	0.05	0.05	-0.3	-0.04	0.07	-0.12	0.01	-0.01
	(-0.74,0.26)	(-0.15,0.13)	(-0.01,0.09)	(-0.07,0.09)	(-0.04,0.14)	(0.04,0.07)	(-0.55,-0.06)	(-0.30,0.23)	(-0.06,0.19)	(-0.28,0.04)	(-0.16,0.19)	(-0.05,0.03)

[a] All associations were adjusted for child sex, race/ethnicity, preterm birth, asthma, infections, corticosteroid episodes and clinical encounters at 0–47 months of age.
[b] Covariates contemporaneous with the exposure periods (corticosteroids, infections and clinical encounters) were adjusted for age-specific antibiotic exposures.
[c] Additionally adjusted for previous antibiotics exposure periods.

© 2021 Aris IM et al. *JAMA Network Open*.

eFigure 1. Scatterplot of Standardized Residuals

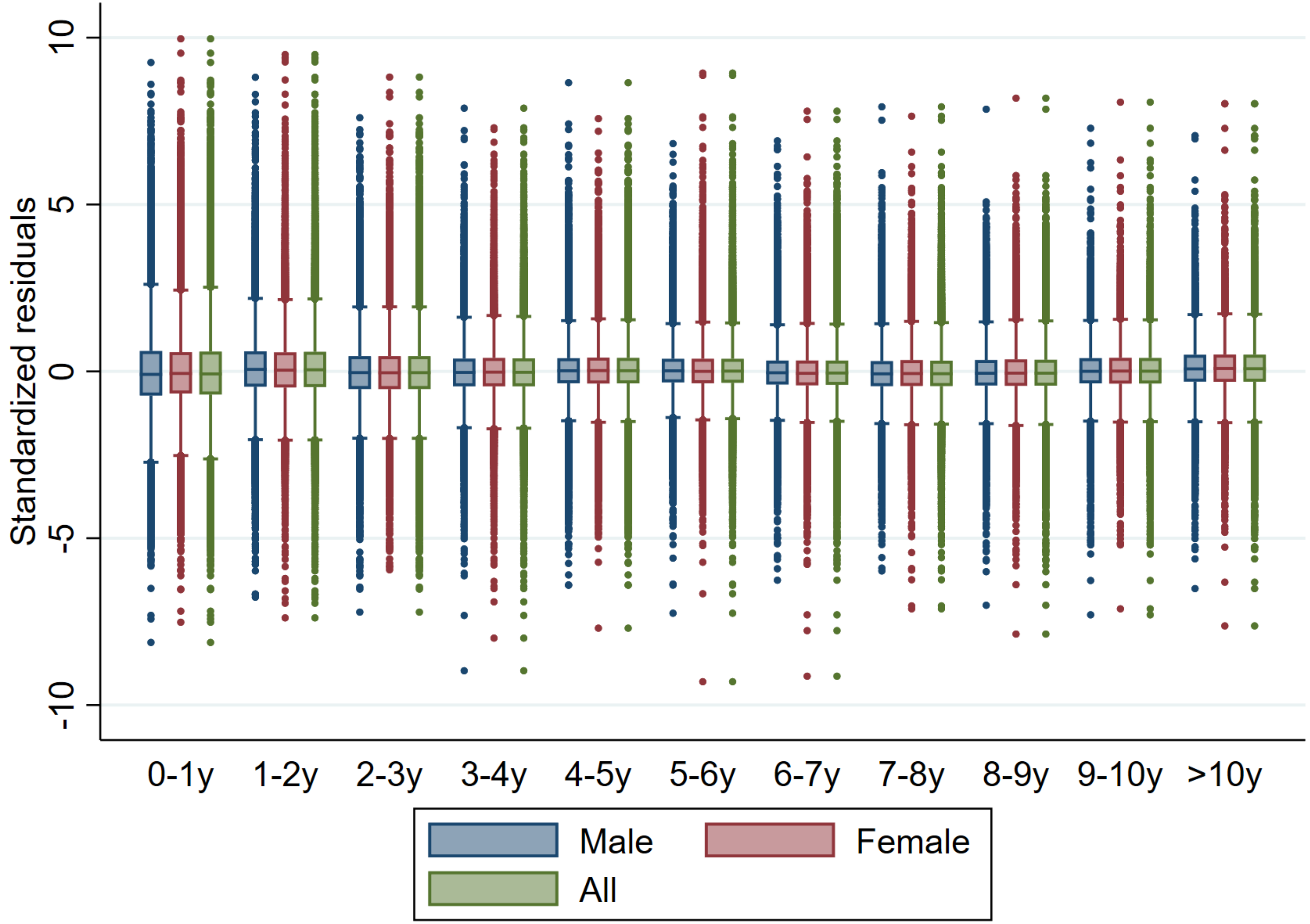

© 2021 Aris IM et al. *JAMA Network Open*.

eFigure 2. Associations of Antibiotic Exposure at 0-5 Months With Age and Magnitude of BMI at Peak, After Excluding Data From 5 Network Partners With the Most Atypical Prescribing Rates

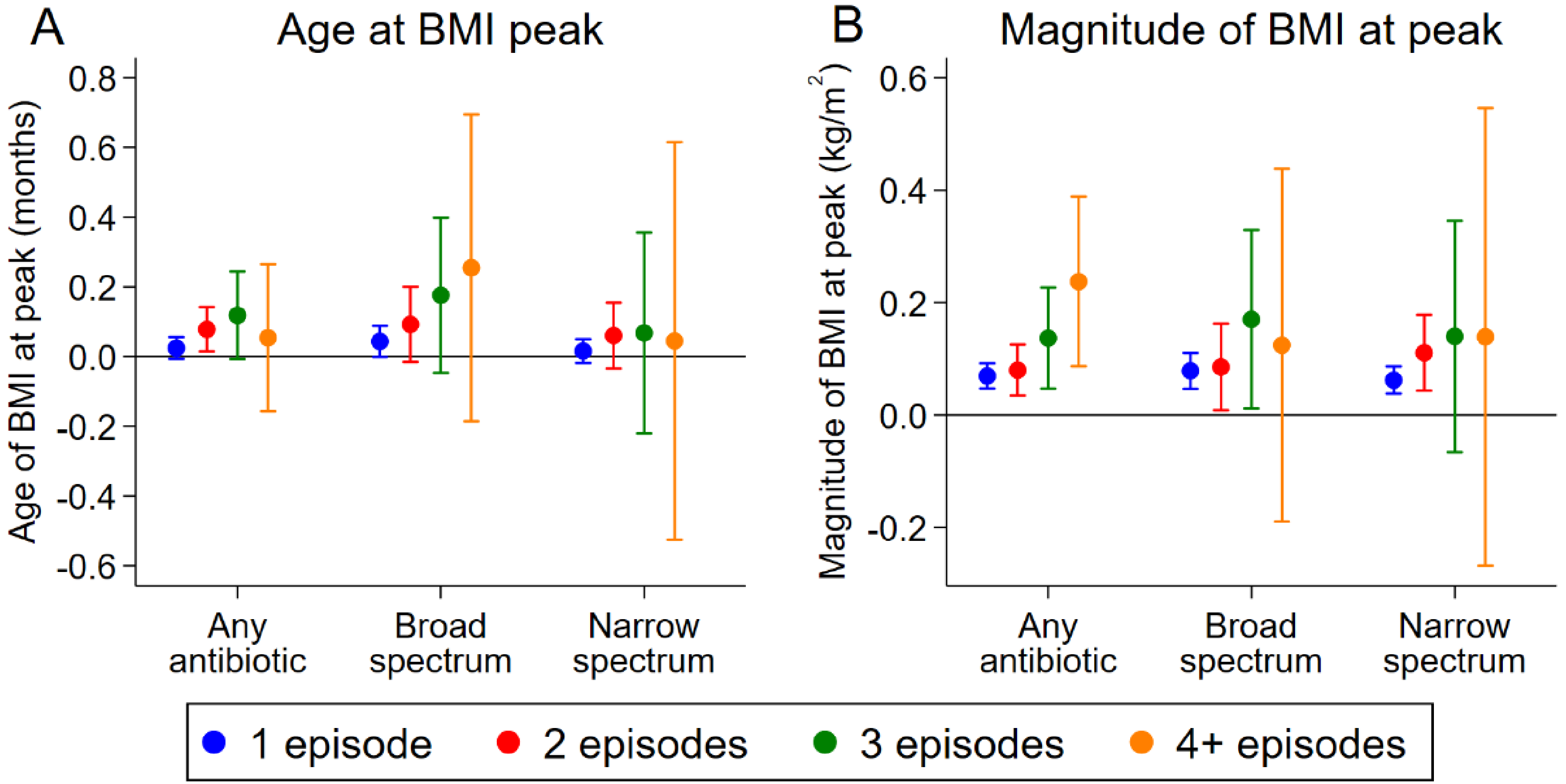

© 2021 Aris IM et al. *JAMA Network Open*.

eFigure 3. Associations of Antibiotic Exposure at 0-47 Months With Age and Magnitude of BMI at Rebound, After Excluding Data From 5 Network Partners With the Most Atypical Prescribing Rates

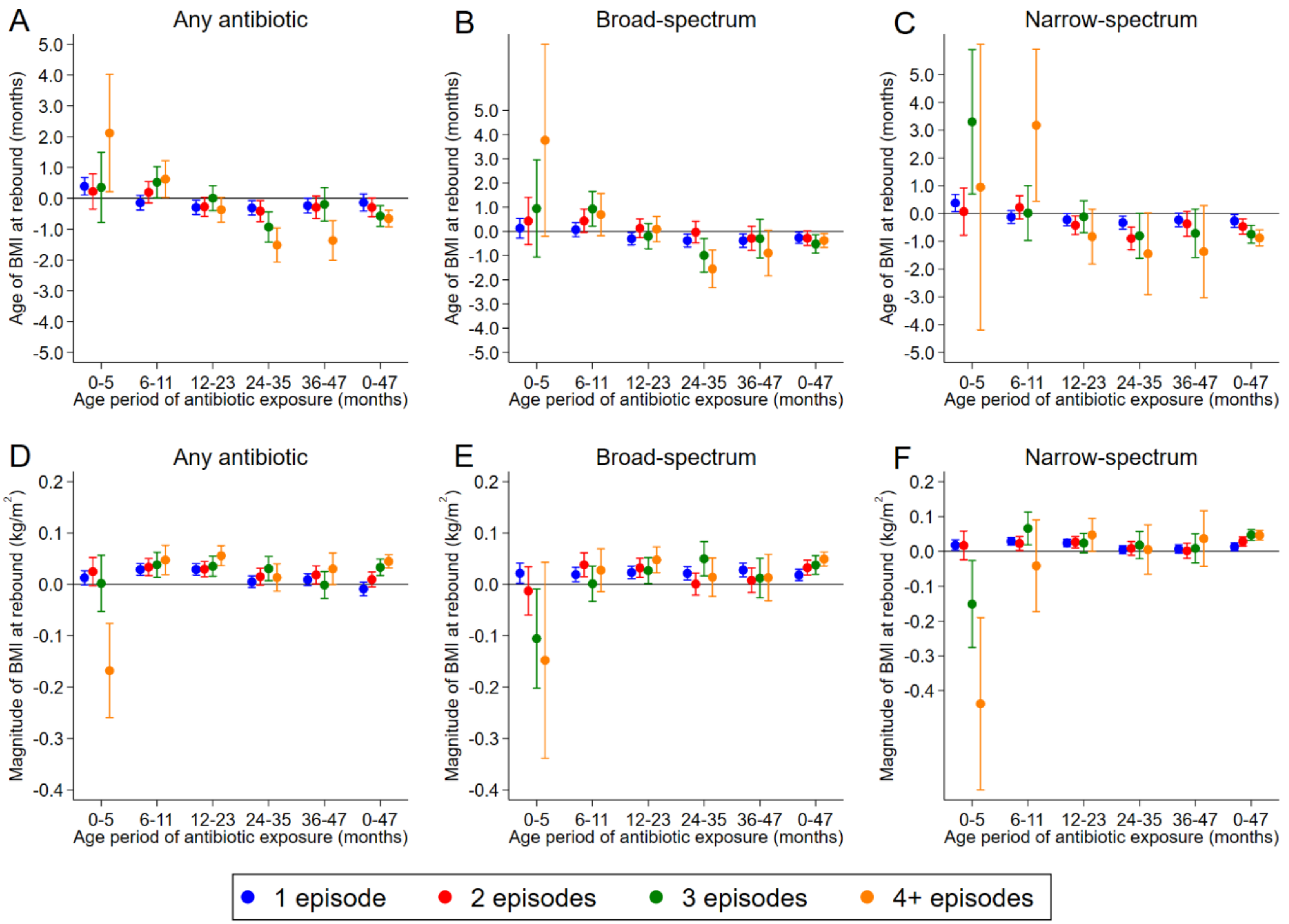

© 2021 Aris IM et al. *JAMA Network Open*.
